# An Observational Study to Assess Postoperative Pain Control and Formulate a Comprehensive Approach to the Implementation of Policy Change for Pain Control in Postoperative Units

**DOI:** 10.7759/cureus.33026

**Published:** 2022-12-27

**Authors:** Gowtham Sundaram Venkatesan, Sri Vishnu Thulasiraman, Balaji Kesavan, Nithyapriya Chinnaraju, Elangkumaran V Manoharan, Priyanga Kesavan

**Affiliations:** 1 General Surgery, James Cook University Hospital, Middlesbrough, GBR; 2 Anaesthesiology, University Hospital of North Tees, Stockton-on-Tees, GBR; 3 Ophthalmology, Kovai Medical Center and Hospital (KMCH) Institute of Health Sciences and Research, Coimbatore, IND; 4 General Medicine, Kovai Medical Center and Hospital (KMCH) Institute of Health Sciences and Research, Coimbatore, IND; 5 Dentistry, Kovai Medical Center and Hospital (KMCH) Institute of Health Sciences and Research, Coimbatore, IND

**Keywords:** multimodal pain management, postoperative pain management, postoperative pain control, implementation of change of policy, change of practice, quality improvement projects, acute pain management, acute pain

## Abstract

Background

Postoperative pain control irrespective of the magnitude of surgery has always remained a challenge for clinicians and healthcare workers. Good postoperative pain control is pivotal for unremarkable recovery and shorter hospital stays. Unfortunately, there is no uniform approach across the globe to address postoperative pain control. This provoked our thought to conduct a prospective observational study in our center to assess the already existing efficacy of pain management.

Materials and methods

This is a prospective observational study conducted in a tertiary care center in Coimbatore, India. The aim of this study is to assess the efficacy of an ongoing pain management system to compare it with standards in the literature to introduce changes and re-examine the results. A total of 100 patients who underwent major surgical procedures from various specialities were included after satisfying the inclusion criteria. The study was conducted over a period of four months to collect data from patients in the postoperative ward. Data were collected, pain-related variables were tabulated, and deficits were identified. Standardized pain assessment tools were not used. The results suggested the need for a policy change for quality improvement. This article gives reports on initial study results and plans to address the deficits in the current pain management system. A systematic and schematic approach for the implementation of the policy change and the framework for the new acute pain service team aiming at quality improvement have been discussed in detail.

Results

The results show that 28 patients were prescribed only routine paracetamol and rescue nonsteroidal anti-inflammatory drugs (NSAIDs). At rest, 56 patients had some pain, and 29 complained of moderate to severe pain. On movement, only seven patients had no pain, 48 had mild pain, and 45 had moderate to severe pain. Only 12 patients out of 100 had good sleep, 27 had moderate, and 43 had little sleep. Twelve patients had no sleep due to continuous ongoing pain in spite of ongoing pain control modalities. Sixteen patients complained of undue delay in receiving their analgesics. Twenty-two patients were dissatisfied, and 44 suggested the need for improvement of current pain control strategies. These data clearly suggest that the pain control strategies are inadequate and need improvement undoubtedly for quality improvement. The Wendy Hirsch model is chosen to create a framework for implementing a new change, and a detailed report is done to present to the hospital quality control department. These changes will be done after the approval, and a post-implementation outcome will be studied.

Conclusion

Good postoperative pain control is of paramount significance for both patients and healthcare professionals. With the current availability of various pain relief modalities, one should consider establishing a pain control pathway, if possible an acute pain team with a systematic approach. These measures not only improve patient satisfaction but also improve postoperative outcomes and better ways of utilizing healthcare resources.

## Introduction

Postoperative pain after major surgeries such as laparotomy needs a multimodal approach for patient comfort, complication prevention, early ambulation, rehabilitation, recovery, and discharge [1,2]. Most hospitals do not have uniform policies on postoperative pain control and are driven by clinician-based decisions on a patient-to-patient basis. This strategy may not be helpful unless there is a round-the-clock acute pain service in the hospital to address the patient’s individual needs. Improper planning of pain management in the postoperative period often leads to inadequate pain control and patient discomfort on one end or an overdosed patient with many analgesics and related complications on the other end [2]. Inadequate pain control is associated with patient complications such as increased sympathetic activity, risk of major cardiac events, poor mobilization, venous thrombosis, atelectasis, wound infection, chronic pain syndrome development, longer hospital stay, and poor patient experience [2].

The Faculty of Pain Medicine from the United Kingdom published core standards for pain management services in 2021, which state that all patients with acute pain must have an individualized plan for pain control appropriate to their clinical condition that is effective, safe, and flexible with a review at frequent intervals [3]. It also states that all inpatients with acute pain must have regular pain and functional assessment using consistent and validated tools, with results recorded.

Any deficiency in patient care is not evident until it is studied with valid methods such as patient questionnaires, feedback, audits, or statistical studies. A detailed review of the literature shows that there is no ideal system that is flawless and acceptable to all settings [2]. Every hospital should have its own policy on pain control, which suits their patient’s spectrum, surgeries performed, availability of resources, and expertise of the doctors and staff in the postoperative ward [3,4].

We did a prospective observational study in our hospital to assess the efficacy of pain management, associated complications, and patient comfort score in postoperative patients. The results warranted establishing a new policy for pain management in this group of patients. Under the guidance of a team of experts, recommendations have been put forward for an initial experiment. A pragmatic approach to the implementation of suggested changes has been made in a stepwise manner [5]. The results have been striking, with improved patient outcomes. The comparison of the pre- and post-implementation of the change was beyond the scope of this article, and this article only summarizes the deficiencies in the pain control policies and the suggested comprehensive approach to the implementation of policy change in the postoperative units.

## Materials and methods

The study is a single-center prospective observational study done in a tertiary care regional-level referral hospital in India. A total of 100 patients who underwent laparotomies in various specialities were included. Approval from the Kovai Medical Center and Hospital (KMCH) Ethics Committee was obtained to undertake the study (approval number: ECR/112/Inst/TN/2013). No new interventions were done. Data were collected from the patient files and feedback forms rather than from the patients themselves. Consent from the medical director and medical records department was obtained to collect these data. None of the patients’ personal details were recorded. The inclusion criteria were defined by any patient having an open laparotomy for surgical access. Patients between 18 and 80 years old were included in the study as the extremes of age will have confounding factors in assessing pain. Patients with chronic pain syndromes and disseminated cancer requiring palliative surgery are excluded from the study. Patients having relook laparotomies are excluded for the risk of duplication of data. The anesthetic doctors and surgical doctors were blinded for the study as this might make the clinicians more vigilant, and so, they might tend to prescribe more analgesics than their routine, which will cause a potential bias.

A data collection sheet is designed prior to conducting the study to include patient demographic details (random allotment number to avoid patient name) such as age, sex, comorbidities, history of chronic pain syndromes, use of any pain modulators, any contraindications for any analgesic technique (such as nonsteroidal anti-inflammatory drugs (NSAIDs) in renal dysfunction), drug allergy to analgesic medications, details of the surgery (actual procedure performed, incision type, duration of procedure, and intraoperative and postoperative analgesic strategies adopted), postoperative pain control variables (pain score at rest, pain score on movement, and overnight sleep), and complications due to pain control medications (sedation that precludes physiotherapy, postoperative nausea and vomiting (PONV), respiratory depression, and hypotension requiring treatment).

Data were collected between December 2016 and March 2017 to include the first 100 patients from the start date who satisfy the inclusion criteria. Data were recorded into an Excel sheet (Microsoft Corp., Redmond, WA, USA) for further analysis. Data were then analyzed and interpreted in the context of the efficacy of pain management for assessing the standard of quality against the standards laid by the Faculty of Pain Medicine [3]. The pictorial representation of data using bar charts and pie diagrams was made and presented in the hospital quality control meeting. The chairpersons included representatives from hospital management, surgical team lead, anesthetic team lead, senior nursing officers, nurses from the postoperative ward, physiotherapists, and quality control board officers. The results showed that existing pain control strategies were not sufficient to yield a better patient satisfaction score. Recommendations were proposed for changes in the existing pain control policies with a standardized tool that gives a qualitative approach to implementing the change. This included domains such as creating the implementation team (acute pain service team), uniform established protocol for all patients, desired outcome variables, roles and responsibility allocation, training and coaching, plan and infrastructure, and post-implementation evaluation [6,7].

The proposed change was agreed upon by the team and gained acceptance among the postoperative care providers at various levels: doctors, nurses, physiotherapists, and patients. This article gives a concise report on the initial study results, the deficits in the pain control policy, and the schematic approach for implementing the suggested change of policy aiming at quality improvement.

## Results

The demographic details of the patients were recorded. The mean age of the patients and sex ratio were also recorded. The patients were broadly classified based on specialities to include representation from all different types of laparotomies as they were the patients cohorted together in the postoperative ward. This data could not be subjected to subgroup analysis as the sample size in the individual groups was less to undergo a univariate analysis. General surgery, colorectal surgery, hepatobiliary surgery, endocrine surgery, urology, gynecology, and surgical oncology are the specialities included. The type of incision might have an influence on pain quality and severity. The type of incision is also included in data collection, again for the fact that it represents all different types of laparotomies. The demographic details of the included patients are shown in Table 1.

**Table 1:** Demographic details SD: standard deviation

Demographic details	Number of patients (number (%))
Sample size (number)	100
Age (in years) (mean ± SD)	49.45 ± 13.72
Male:female	63:37
Mean duration of the procedure (in minutes)	246 ± 98
Speciality	
General surgery	24 (24%)
Colorectal surgery	22 (22%)
Hepatobiliary surgery	6 (6%)
Endocrine surgery	5 (5%)
Urology	15 (15%)
Gynecology	18 (18%)
Surgical oncology	6 (6%)
Incision types	
Midline	44 (44%)
Subcostal (unilateral)	16 (16%)
Rooftop (subcostal bilateral)	8 (8%)
Paramedian (all incisions other than midline)	21 (21%)
Pfannenstiel incision	11 (11%)

The previously existing pain management strategies were clinician-based and differ from patient to patient undergoing similar surgery. The analgesic plans were regional nerve blocks (most commonly transversus abdominis plane (TAP) block), intravenous (IV) analgesics (paracetamol, NSAIDs, and rarely opioids), transdermal fentanyl patches, and epidural analgesia with continuous infusion. These strategies were used either as a sole technique or in combination. Almost all patients received intravenous (IV) paracetamol round the clock with rescue analgesics such as diclofenac or ketorolac. The pain-related variables are listed in Table 2.

**Table 2:** Pain-related variables NSAIDs: nonsteroidal anti-inflammatory drugs; TAP block: transversus abdominis plane block; PCA: patient-controlled analgesia; IV: intravenous; PONV: postoperative nausea and vomiting

Postoperative pain-related variables (number (%))	
Type of analgesic used (over routine paracetamol and rescue NSAIDs)	
Fentanyl patch	58 (58%)
Epidural infusion	6 (6%)
Regional nerve block (TAP block)	8 (8%)
PCA opioids via IV infusion	0 (0%)
None	28 (28%)
Overnight pain score at rest	
No pain	15 (15%)
Mild pain	56 (56%)
Moderate to severe pain	29 (29%)
Overnight pain score on movement	
No pain	7 (7%)
Mild pain	48 (48%)
Moderate to severe pain	45 (45%)
Overnight sleep	
Good	18 (18%)
Moderate	27 (27%)
Little	43 (43%)
No sleep due to pain	12 (12%)
Overall patient satisfaction score	
Satisfied	34 (34%)
Satisfied but needs improvement in pain control	44 (44%)
Dissatisfied	22 (22%)
Complications due to analgesics	
Hypotension requiring treatment/warrants epidural infusion to stop	4 (4%)
Drowsiness precluding physiotherapy, oral feeds	22 (22%)
PONV requiring treatment/causing delay in starting oral feeds	12 (12%)
Episodes of respiratory depression due to any form of analgesics	4 (4%)
NSAID-related renal dysfunction	5 (5%)
Number of patients complained about delays in receiving analgesics	16 (16%)

The results from Table 2 show that 28 patients were prescribed only routine paracetamol and rescue NSAIDs. There were no plans in place if the NSAIDs did not address the pain or if they cannot be given for some reason. Patient-controlled analgesia is a better mode of pain control as it avoids overdosing on opioids, is readily available and avoids delay between the patient feeling the discomfort and receiving the painkillers, has better patient acceptance and satisfaction as the patients themselves have control of their pain, and results to decreased workload to the clinical staff. Evidence suggests that the patient-controlled analgesia (PCA) mode is superior to the “pro re nata” (PRN) (which means as and when needed) mode of drug prescription in quality of pain control, safety, and patient acceptance [2,8,9]. However, none of the patients in our study had PCA mode as the practice of PCA has not been introduced then.

Transdermal fentanyl patches are used by more than half of the patients and have been useful. Only six out of 100 patients received an epidural, revealing the fact that the anesthetic population is moving away from central neuraxial techniques due to the potential complications associated with it, particularly when there are safer alternatives available [9-12]. Even out of the six epidural infusions, four were stopped due to hypotension.

Fifteen patients had no pain at all at rest, while 56 had some pain and 29 complained of moderate to severe pain. On movement, only seven patients had no pain, 48 had mild pain, and 45 had moderate to severe pain. The movement may be for positioning on the bed, rolling over in the bed, mobilizing to a chair, ambulating for physiotherapy, mobilizing to the toilet, etc. Only 12 patients out of 100 had good sleep, 27 had moderate sleep, and 43 had little sleep. Twelve patients had no sleep due to continuous ongoing pain in spite of ongoing pain control modalities.

Sixteen patients complained of undue delay in receiving their analgesics. This could be possibly because of poor nurse:patient ratio or unavailability of any rescue prescriptions and the time consumed to contact a doctor for prescriptions due to being out of hours. Twenty-two patients were dissatisfied, and 44 suggested improvement of current strategies.

## Discussion

The study included all types of laparotomies and most of the common incision types. The representation seemed adequate from a variety of patients that would usually get admitted to the postoperative ward on a daily basis.

An ideal hospital system for postoperative patient care should aim at zero patients with severe pain, predefined plans for pain control particularly to facilitate mobilization and physiotherapy, adequate patient comfort including good sleep, and no PONV or other symptoms related to inadequate or overdoses of painkillers [8,9]. This is challenging to achieve, given the patients’ variability in their physiological status, the nature of pathology, the extent of surgery, their pain tolerance, and variable responses to painkillers, particularly opioids. In spite of these challenges, every system should work toward better delivery of postoperative care [9].

These data clearly suggest that the pain control strategies are inadequate and need improvement undoubtedly for quality improvement when compared to the standards set by the Faculty of Pain Medicine [3]. The core standards laid by the Faculty of Pain Medicine from the Royal College of Anaesthetists, United Kingdom, give recommendations for an ideal working system of pain control in a hospital setting [3].

New pain management involved the formation of an acute pain team, regular monitoring and documentation of pain, escalation when needed, encouraging the use of regional techniques, patient-controlled analgesia (PCA) techniques, etc.

Existing ward practice

The postoperative ward had general ward units with eight beds in a room and units that have two patients in a room and single-patient rooms. The ward capacity can accommodate a maximum of 40 patients at a time. The ward had a dedicated nurse-in-charge and senior and junior nursing staff and nursing assistants around the clock taking care of patients. The nurse:patient ratio is 1:8 to 1:10. One junior medical officer is available to address the key issues of the patient immediately but will be floating across other wards on the floor. The anesthetic and surgical resident doctors are available in-house whenever their advice is needed. They may be caught up in overnight emergency surgeries, which might cause some delay in advice or patient review if needed. Doctors visit the postoperative patients in the ward the next day morning after the surgery. Postoperative orders are given along with operative notes before the patient leaves the theater. This has been the routine practice before the study.

Quality control meeting

The hospital conducts regular audit meetings, quality control meetings, and patient safety meetings as a part of institutional policy. The results from the abovementioned study regarding the current levels of acute postoperative pain services in the hospital and the deficiencies where current practice modification is required were discussed, and recommendations were laid on the implementation of policy change in acute pain services. The meeting included the audit committee members, anesthetic and surgical doctors, postoperative ward nurses, matrons, physiotherapists, quality control board directors, and stakeholders of the hospital. The list of recommendations is suggested and submitted for approval from the quality control and the hospital management team.

The summary of the recommendations can be found in Table 3. The implementation of the policy change has been suggested to take a systematic and structural pathway [13,14]. For a systematic approach to organizational change, the Wendy Hirsch model of change implementation framework is adopted [15].

**Table 3:** Recommendations by the audit committee PCA: patient-controlled analgesia

Recommendations by the audit committee to the quality control department and hospital management
Change of current physician-driven pain control policies
Implementation of current pain control policy change to take a systematic and structural pathway
Establishment of dedicated acute pain management services available around the clock led by the anesthetic team
Pain should be considered as the fifth vital sign, as widely accepted now; the assessment of pain should include subjective and objective variables that will be recorded and scored
Monitoring and objective measurement of pain using standardized pain assessment tools
Training the hospital staff for safe PCA prescriptions and delivery
Improving the infrastructure by procuring more ultrasound machines for nerve blocks and PCA machine
Training of anesthetic doctors for epidural and ultrasound-guided nerve blocks
Regular formal and informal teaching sessions for pain management among ward doctors, nurses, and physiotherapists
Ensuring adequate manpower such as nurse:patient ratio and doctor:patient ratio in the postoperative ward
Regular audits for monitoring the outcome of change

A comprehensive approach for change of practice to construct a local hospital-based pain management service

**Figure 1:**
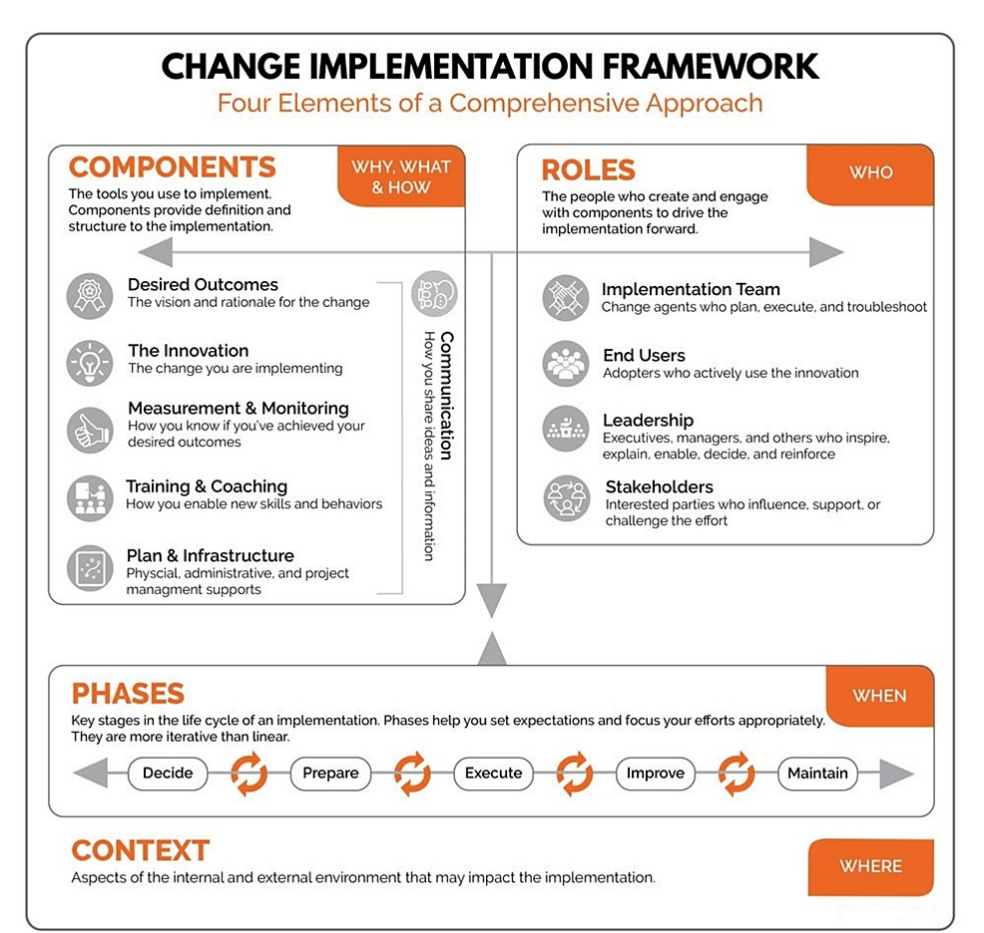
Change implementation framework Published with permission from https://wendyhirsch.com/

The framework for change implementation is shown in Figure 1. Rather than a fixed, step-by-step process or method, this change management framework reflects that successful change is both an art and a science [13]. It clarifies the key elements, and change efforts should include understanding that how you execute them will depend on the type and scale of your change and your organizational context.

Components (why, what, and how)

Components are the tools and techniques to be used to implement a change. Components provide structure, being the backbone of the framework [6,16].

The “Desired Outcome” or the “vision” is obviously a quality improvement in acute postoperative management. The rationale and need for the change came from the quality control team to address patient feedback on previous postoperative pain control.

The “Innovation” of the framework was targeted at the standards laid by the Faculty of Pain Medicine affiliated with the Royal College of Anaesthetists, United Kingdom, to be the criterion standard for designing the new pain management service in the institution. The Faculty of Pain Medicine published the standards and guidelines/recommendations on pain control. The first edition in 2015 has been revised, and recently, the second edition was published in 2021 with recent updates to pain medicine [3].

**Figure 2:**
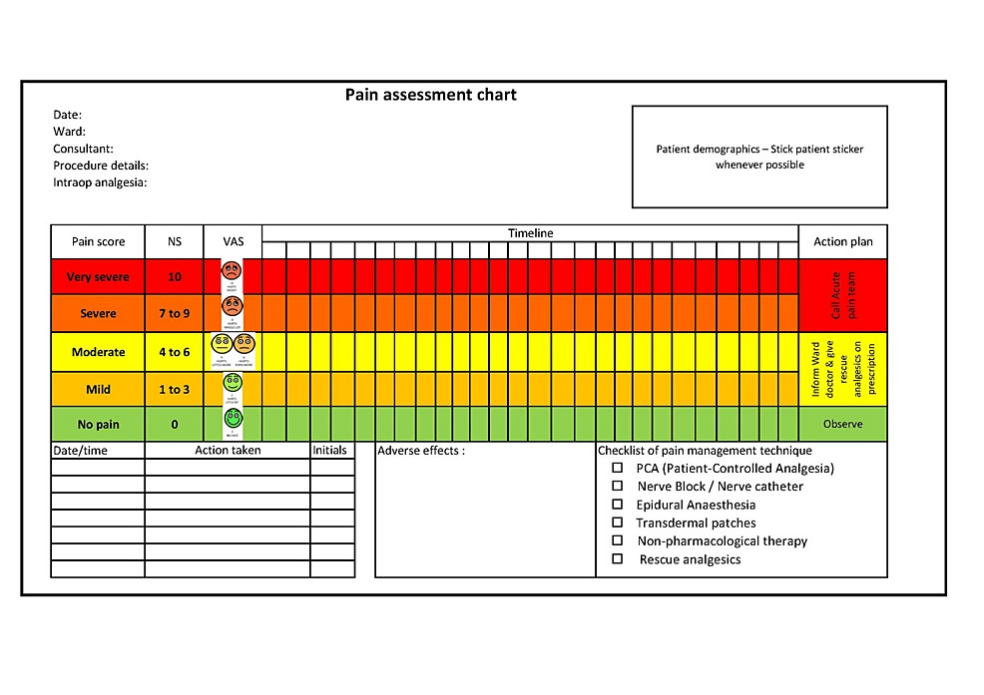
Template for pain assessment chart to be used in the ward NS: numerical score; VAS: visual analog scale Image credits: Gowtham Sundaram Venkatesan

“Measurement and Monitoring” should be approached at two levels: from the patient perspective and the institutional level. Pain should be considered the fifth vital sign, as widely accepted now. There should be subjective and objective variables that will be documented at hourly or two-hourly intervals at least for the first postoperative day. The use of validated pain assessment tools such as the visual analog scale, numerical rating scale, and verbal rating scale should be encouraged to avoid disparity and ambiguity [17]. Pain should be scored, and the definitive targets should be assigned, below which there should be a rescue plan that is accessible [3]. A responsible clinician should be assigned, most often the anesthetist, who is readily available around the clock to address the pain [3,18]. A template for pain assessment at bedside use has been proposed by the audit team to the quality control department. This template is shown in Figure 2.

It is advisable that the anesthetist assigned to the pain team, particularly during out of hours, is trained and has the capacity to perform the common regional nerve block under ultrasound guidance without supervision and is competent to assess, prescribe, administer, and monitor other techniques such as epidural and PCA. From an institutional point of measurement of targets, the quality control department should assess the outcome change from a broader perspective, collect data from medical records to analyze any improvement even if it is minimal, conduct regular audits, regular reports, and meeting from the acute pain team, analyze patient feedback forms, and suggest any further improvement [6].

“Training and Coaching” involves regular continued medical programs on pain and regular formal and informal teaching for ward nurses, ward doctors, matrons, anesthetists, and surgeons. The scope of teaching will include a broader context of pain control such as understanding the pain pathway, assessing severity, troubleshooting, and managing the pain and complications associated with it [16,17]. Other modes of knowledge sharing such as PowerPoint presentations, panel discussions, debates, workshops, and quiz programs should be encouraged. Hospital management should allocate working hours as a part of the teaching schedule at least once or twice a month. Techniques of rewards such as the star of the month and the best performer award should ignite an interest in the staff involved in the direct care of patients [13].

“Infrastructure” is one of the important components without which any implementation change should fail [13]. Specialist acute pain management advice and intervention should be available at all times to all inpatients. Staffing should be sufficient to provide prospective cover for all personnel. This includes adequate staffing from ward nurses, matrons, duty ward doctors, anesthetist resident, and consultants. The nurse:patient ratio should be 1:3 to 1:4 at all times around the clock. The equipment for pain management techniques such as ultrasound machines, PCA delivery devices, and pharmacy including controlled drugs such as opioids, epidural kits, pumps, nerve block kits, and continuous pumps for nerve catheters should be readily available and meet the quality control requirements. These devices should be regularly serviced as per manufacturer recommendations and quality checks passed. Ensuring excess device availability in case of increased demands or device malfunction is advised [9]. There should be reporting system available to report any concerns in the infrastructure. The acute pain team lead should take responsibility for ensuring good infrastructure available liaising with the hospital management [8].

“Communication” is an integral part of the change implementation framework. Communication should be multiway, clear, concise, and objective, use empathy, cultivate confidence, and be anonymous if required. The acute pain team should have a permanent mobile contact facility such as a bleep or phone available around the clock, and everyone in the postoperative ward should be given access to contact the pain service team. Freedom of raising concerns should be ensured, and systems such as Datix should be encouraged to learn from mistakes and build a constructive outlook [16]. A written form of communication is better than oral orders. It is not only the prescriptions or plans but also every single communication need to be documented clearly regarding the questions raised and the solutions offered. The acute pain team record should have empty pages to document these. A proper communication tool and documentation make everyone accountable and more responsible.

Roles (who)

People play various distinct roles to “make the change happen” in an organization, including end users, leadership, management, or part of an implementation team. Role allocation is one of the key factors that leads to the target achievement in any process [19,20]. Roles and responsibilities should be clear.

The “Implementation Team”shall be named as the acute pain team in this context. This team is set up at three levels of hierarchy. The executive arm of the acute pain team should implement the change at the field level. This will include the ward nurses, matrons, physiotherapists, and the ward doctors/medical officers. They are the people who are involved in the direct care of patients. The tasks of this team involve measurement and monitoring of pain, documentation, troubleshooting any complications, and escalation to appropriate seniors of the next hierarchy whenever necessary. Adequate manpower and appropriate formal and informal training will lead to a successful executive team. They should have access to a reporting system, and freedom of expression needs to be ensured for the executive team.

The next level of hierarchy is the managerial arm of the acute pain team, which will consist of the duty anesthetic resident doctor, duty surgical resident doctor, anesthetic consultant on-call, surgical consultant on-call, chief nursing supervisor, and matron of the hospital [8,15]. The task of this team is to formulate a working plan for every single patient before he/she gets admitted to the postoperative ward. The need for pain control management strategies shall be unique for every single patient and tailored to the need. This team ensures the choice of drugs, mode of administration, and need of other procedures such as nerve blocks, epidural, administration of PCA, and dose modification of painkillers as required. A healthcare record or document should be designed, which gives a formulated plan for that patient. This should also include clear prescriptions, documentation of specific complications expected, and when to seek help. It is recommended and also a good medical practice that a representative of this team does a postoperative ward round on every single patient in the evening to ensure that the plans are documented and to address any key concern on pain management [13]. It is emphasized that the morning grand ward rounds should also include the review of the adequacy of overnight pain control and escalating or weaning of pain control strategy and medications.

The “Leadership” team is the top hierarchy in the acute pain team [2,21]. This team is led by the clinical lead or the head of the anesthetic department and the medical director. The lead supervises the ongoing process for its effective functioning. He liaises with the anesthetic nurses and on-call consultants for ensuring that pain is addressed in the right way and the right time. He is expected to conduct a weekly meeting to get reports of the pain management work and goes through the pain management register. He also acts as the bridge between the executive team and the managerial team on one side and the stakeholders on the other side for any improvement, facilitation, or infrastructure. The decisions beyond the capacity of the lead such as planning a new infrastructure may need authorization from the medical director of the hospital and approval from stakeholders.

The “Stakeholder” in this context would refer to the hospital management. The management, with views of maintaining delivery of good quality medical service to the community, should review the reports from the authorities and encourage audits on pain control and conduct regular training sessions to develop a positive and constructive outlook on patient feedback to enhance the quality. The infrastructure should be excellent, and it is the stakeholder’s responsibility to ensure this quality [22,23].

**Figure 3:**
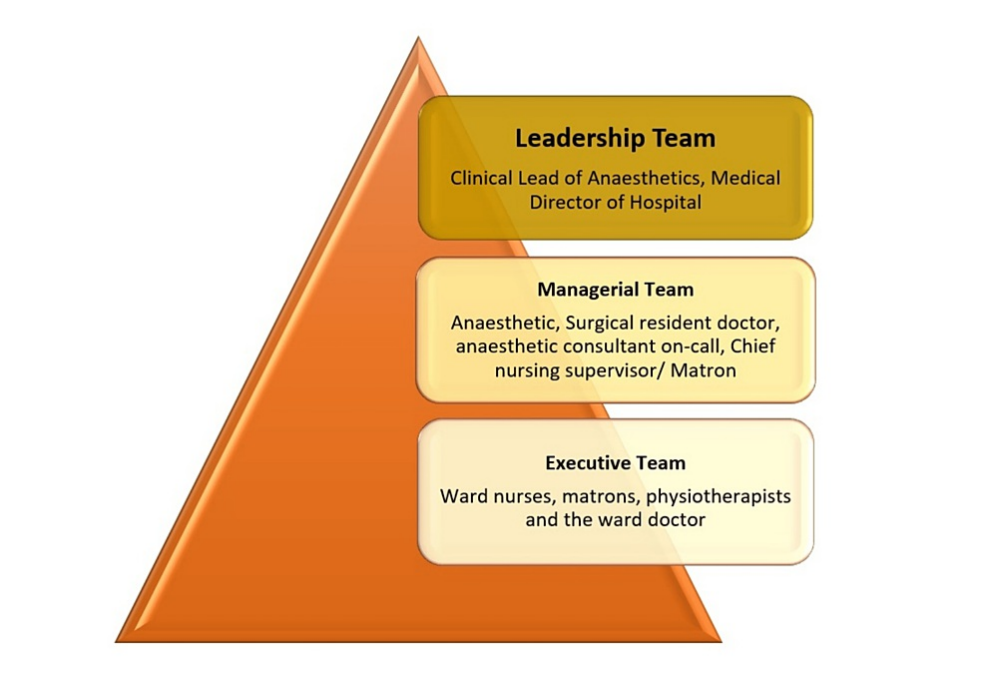
Hierarchy of the acute pain team Image credits: Balaji Kesavan

“End Users” in this context will be the postoperative patients. In another context, everyone from the hospital enjoys the benefits of a better pain management. The nurse taking care of the postoperative patient and the doctor in charge of the ward will be able to discharge their duties better and have peaceful shifts, the anesthetic resident gets training, and the hospital gets a good reputation [15,21]. The hierarchy of the acute pain team is shown in Figure 3.

Phases (when)

Effective change management process is a multiphased, iterative effort rather than a straightforward, linear progression from “start” to “finish.” Thinking of it as such better enables the planners to focus on the right things at the right time. It also helps set appropriate expectations for what is expected to happen when and at what speed [7,16].

According to the Wendy Hirsch model of change implementation framework, the phase is a process of continuous steps but can go in either direction [15]. The phases are reversible and flexible to go back and forth for a constructive and positive outcome [24]. The phases can be divided into Decide, Prepare, Execute, Improve, and Maintain as shown in Figure 1.

The acute pain management team, once approved, will be decided, and a framework will be prepared. The “Execute,” “Improve,” and “Maintain” phases are to be expected. However, these phases need to be observed with targeted outcomes such as improvement in patient satisfaction score, reduction of complications, and reduction in the incidence of pain complaints. The data should be collected and audited to be compared with the baseline. Any improvement is appreciated, any unwarranted change can be withdrawn, and further changes can be suggested and made. Cost-effective analysis also needs to be done at the “Execute” or “Improve” phase [8,25].

Context (where)

Context is the system or where the change is to be implemented, and outcomes will be observed [26]. Since the root cause or the rationale behind this change implementation has come from the postoperative surgical ward, this acute pain management team will deliver care in the same ward. Based on the outcomes, they will be extended to the palliative care ward, trauma ward, postoperative cardiothoracic and neurosurgical units, and burns ward, where acute pain control is very much necessary.

Proposal of policy

This schematic proposal was submitted by the audit committee to the board of quality control members and the hospital management. This was considered by them for approval and has been in practice with various post-implementation changes in the next few years. The results are being collected and will be compared with the pre-implementation data for quantifying the success of the policy change. This report aims at giving a schematic approach to designing an acute pain team in a regional center hospital adopted from core standards published by the Faculty of Pain Medicine of the Royal College of Anaesthetists [3].

Limitations

This is a single-center experience with a low volume of patients. The post-implementation study is not discussed in this study as it is beyond the scope of this article. Although this implementation plan would help a tertiary care hospital with a large volume of patients, smaller hospitals with resource-limited settings might not be able to implement this approach effectively.

## Conclusions

Adequate pain control is one of the basic rights of patients and one of the basic duties of physicians/surgeons, particularly in the postoperative period. Modern medicine and techniques give us a variety of pain control strategies that are safe and effective. A systematic and strategic approach to the implementation of a pain control policy will be the most efficient means of generating consistent and optimum results. This report will be a useful tool or guide for setting up an acute pain service team in larger hospitals.
